# Effects of Particle Size and Surface Chemistry on the Dispersion of Graphite Nanoplates in Polypropylene Composites

**DOI:** 10.3390/polym10020222

**Published:** 2018-02-24

**Authors:** Raquel M. Santos, Sacha T. Mould, Petr Formánek, Maria C. Paiva, José A. Covas

**Affiliations:** 1 Institute for Polymers and Composites/I3N, University of Minho, Campus de Azurém, 4800-058 Guimarães, Portugal; rmsantos@inegi.up.pt (R.M.S.); sacha.tm@dep.uminho.pt (S.T.M.); 2 Leibniz Institute for Polymer Research Dresden, Hohe Strasse 6, D-01069 Dresden, Germany; formanek@ipfdd.de

**Keywords:** graphite nanoplates, surface modification, polymer composites, dispersion

## Abstract

Carbon nanoparticles tend to form agglomerates with considerable cohesive strength, depending on particle morphology and chemistry, thus presenting different dispersion challenges. The present work studies the dispersion of three types of graphite nanoplates (GnP) with different flake sizes and bulk densities in a polypropylene melt, using a prototype extensional mixer under comparable hydrodynamic stresses. The nanoparticles were also chemically functionalized by covalent bonding polymer molecules to their surface, and the dispersion of the functionalized GnP was studied. The effects of stress relaxation on dispersion were also analyzed. Samples were removed along the mixer length, and characterized by microscopy and dielectric spectroscopy. A lower dispersion rate was observed for GnP with larger surface area and higher bulk density. Significant re-agglomeration was observed for all materials when the deformation rate was reduced. The polypropylene-functionalized GnP, characterized by increased compatibility with the polymer matrix, showed similar dispersion effects, albeit presenting slightly higher dispersion levels. All the composites exhibit dielectric behavior, however, the alternate current (AC) conductivity is systematically higher for the composites with larger flake GnP.

## 1. Introduction

Polymer matrix-based nanocomposites have attracted intensive research and development effort, due to their potential for developing novel, cost-effective, and high-performance products for advanced engineering applications, for example, in aerospace, automotive, construction, and medicine. The development of nanocomposites with high thermal and electrical conductivity, which are normally based on the incorporation of conductive carbon nanostructures (graphite and its derivatives, graphene, carbon nanotubes, and fullerenes) [1,2], motivates particular attention given their promising applications in single molecule gas detection, transparent conducting electrodes, energy storage (supercapacitors and lithium ion batteries), transportation devices, refractory and fire-proof materials, etc. [3,4,5].

Graphite is the most thermodynamically stable and softest (1 in the Mohs hardness scale) form of carbon with high stiffness (1 TPa), and good electrical (>10^3^ S·m^−1^) and in-plane thermal (~2000 W·m^−1^·K^−1^) conductivities [4,5]. Intercalation of graphite by electron donors (alkali metals) or electron acceptors (acids, halogens, and halide ions) allows for increasing the interlayer spacing and weakening the van der Waals interactions, yielding expanded graphite, with particle sizes around 300–500 µm [6,7]. This is a material of high interest for fire protection [8]. When submitted to a critical temperature or microwave radiation, intercalated graphite can expand hundreds of times relative to its initial volume, due to vaporization of the intercalate, forming exfoliated graphite (EG) [9]. In turn, ultrasonication of the latter in specific solvents or surfactant solutions results in stable suspensions (with particle sizes typically lower than 50 µm), usually designated as graphite nanoplates (GnP) [10].

In order to reach its optimal effect as composite reinforcement, graphite should be exfoliated as much as possible into its separate layers. In practice, the effective dispersion of GnP in polymeric matrices remains difficult, not only due to their inherent tendency to form cohesive clusters [11], but also because the chemical inertia of GnP precludes the development of strong interfaces with polymers. Surface modification of the nanoparticles may minimize these limitations. It can be achieved via non-covalent or covalent approaches [12]. Covalent functionalization allows the formation of strong and stable interfaces with polymeric matrices by chemical bonding of functional groups to the aromatic carbon lattice of graphene. The prevalent strategy consists on the chemical oxidation of the graphene surfaces as described by Brodie [13], Staudenmaier [14], and Hummers [15]. However, these procedures disturb the conjugated nature of the graphitic lattice by local bonding of oxygen-containing groups, shifting its hybridization state from *sp*^2^ to *sp*^3^, as indicated by Raman spectroscopy studies [16,17]. Reactions involving the cycloaddition to the π-electrons of the graphene lattice were successfully applied to carbon nanotubes [18,19], carbon nanofillers [20], and graphite nanoplates [3] under mild conditions, leading to little or no damage of their structural integrity.

Currently, it is well accepted that the dispersion of carbon nanoparticles into polymeric matrices and the electrical percolation threshold of nanocomposites are strongly dependent on nanoparticle surface chemistry [3,21,22], agglomerate density and strength [23,24], size [25], aspect ratio [26], purity [27], alignment [28,29], as well as on polymer type [30] and viscosity [31,32,33]. The influence of the majority of these parameters has been well documented for multi-walled carbon nanotubes/polymer composites, while studies with GnP/polymer composites are less abundant [3,11,25,29,34,35,36]. In particular, a thorough understanding of the dispersion and re-agglomeration phenomena of GnP in polymer melts is still lacking. Vilaverde et al. [11] investigated the dispersion and re-agglomeration of 2 and 10 wt % of GnP in polypropylene (PP) during flow under well-controlled conditions. A gradual decrease of the number and size of the GnP agglomerates was observed, regardless of the shear rate, but the magnitude of the changes was highly dependent on graphite concentration. At sufficiently low shear rates, interparticle interactions induced GnP re-agglomeration. Santos et al. [3] demonstrated that chemical modification of GnP via 1,3 dipolar cycloaddition enhanced the stability of dispersion and delayed re-agglomeration.

In this work, the effects of morphology and surface chemistry of GnP on dispersion and re-agglomeration in polypropylene are investigated. Composites containing 2 wt % of GnP with different morphology, as-received or functionalized, were prepared using a prototype small-scale extensional mixer coupled to a capillary rheometer, in order to generate well controlled flows in terms of volumetric rate and temperature. Samples of the composites were collected along the length of the mixer and cooled in liquid nitrogen for subsequent analysis. Dispersion and re-agglomeration were analyzed by microscopy, and the electrical conductivity of the composites was measured.

## 2. Experimental

### 2.1. Materials

Polypropylene copolymer (Icorene CO14RM^®^, from ICO Polymers, Inc., Allentown, PA, USA), in powder form, with a melt flow index of 13 g·10 min^−1^ (190 °C, 2.16 Kg) and a density of 0.9, was used as matrix. Three commercially available graphite nanoplates were obtained from XG Sciences (Lansing, MI, USA), their properties being summarized in Table 1 according to the company data sheets.

GnP were chemically functionalized via 1,3-dipolar cycloaddition (DCA) of an azomethine ylide, as reported elsewhere [18]. The reaction was carried out using *N*-benzyloxycarbonylglycine (Z-GLY-OH) 99% from Sigma Aldrich (St. Louis, MO, USA) and paraformaldehyde reagent grade, from Sigma Aldrich (St. Louis, MO, USA), homogeneously mixing with the GnP in powder form, and maintained at 250 °C for 3 h. The pyrrolidine groups formed at the surface of functionalized GnP were further covalently bonded to polypropylene-*graft*-maleic anhydride (PP-*g*-MA) containing 8–10 wt % of maleic anhydride, supplied by Sigma-Aldrich (St. Louis, MO, USA), to produce GnP covalently functionalized with PP-*g*-MA (*f*GnP-PP). The reaction was performed by refluxing in toluene during 3 h. Extensive washing with hot toluene was carried out to remove the excess PP-*g*-MA, and the remaining material was dried in an oven overnight at 100 °C.

### 2.2. Processing of Nanocomposites

PP nanocomposites containing 2 wt % of as-received GnP or *f*GnP-PP, pre-mixed in powder form, were processed in a small-scale extensional mixer attached to a Rosand RH10 capillary rheometer (Malvern Instruments Limited, Malvern, UK), heated to a predefined temperature, and forced through the system at 50 mm·min^−1^, after preheating for 5 min. This GnP loading and flow rate were selected based on previous dispersion studies of 2 and 10 wt % GnP in PP [3,11], demonstrating that monitoring dispersion and re-agglomeration of nanocomposites containing high GnP concentrations was hindered by the large amount of agglomerates present. The temperature was adjusted so that the total pressure drop, i.e., the hydrodynamic stresses, would remain approximately uniform.

**Figure 1:**
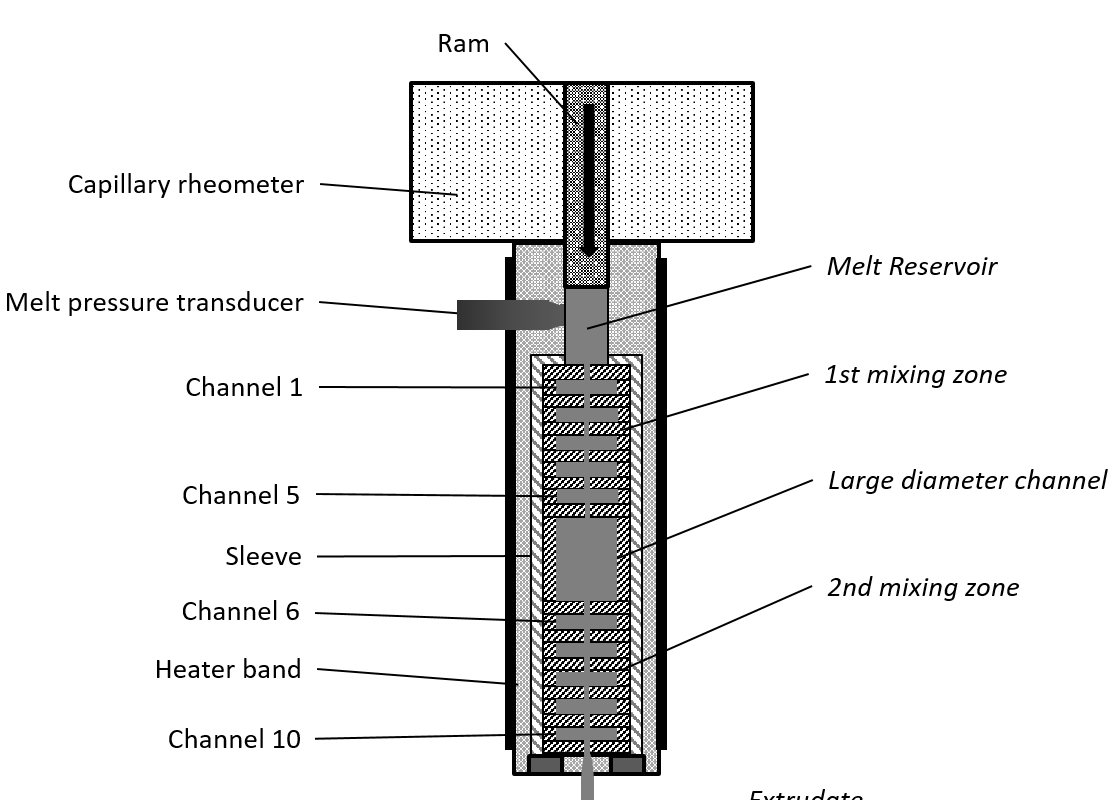
Prototype small-scale mixer, with two mixing zones separated by a large diameter channel. Each mixing zone contains a series of circular channels with alternating diameters, creating sequences of converging/diverging flows. Removing the sleeve and separating the individual rings gives access to material samples for subsequent characterization.

The mixer consists of three main sections (see Figure 1). Initially, the material streams through a sequence of 10 circular channels with alternating diameters, *d* (*d* = 1 and *d* = 8 mm, length = 2 mm) that create a series of converging/diverging flows, with a strong extensional component (this will be denoted as first mixing zone). The average shear rate attains approximately 1500 s^–1^ in the smaller channels. As demonstrated by Grace [37] for liquid suspensions, normal stresses are more efficient for dispersion than shear stresses. Consequently, at the outlet of this first mixing zone, extensive dispersion levels are anticipated. In order to assess the stability of the dispersion achieved, the material enters the second section of the mixer, which contains a larger cylindrical channel (*d* = 18 mm and length = 24 mm) where shear rates are negligible (0.3 s^−1^ in the experiments performed). Stress relaxation occurs, and re-agglomeration could eventually develop. The third section of the mixer is identical to the first one (and denoted as second mixing zone). The aim here is to subject the composite to similarly high deformation rates, and again, induce dispersion. The set-up is of modular construction. It is not only possible to vary the number and geometry of the individual channels, but material samples can be collected from the larger channels (with *d* = 8 and *d* = 18 mm), as well as from the reservoir of the rheometer, cooled in liquid nitrogen, and subsequently characterized. It was shown that the levels of dispersion obtained with this device are comparable to those achieved with the frequently used twin screw extruder [22,38].

### 2.3. Characterization of Graphite Nanoplates

The area of the as-received GnP and *f*GnP-PP primary particle agglomerates was measured by optical microscopy (OM) using a BH2 Olympus microscope attached to a Leica DFC 280 digital camera (Hamburg, Germany). Samples were prepared by hand mixing a small amount of GnP in epoxy resin, spreading a thin layer on a glass slide, and curing it at room temperature overnight. At least 130 optical micrographs were analyzed for each powder type. The morphology of the as-received GnP and *f*GnP-PP was characterized by transmission electron microscopy (TEM), by means of a JEOL (Peabody, MA, USA) JEM1010 equipped with a CCD Orius camera and a tungsten filament as electron source, at an acceleration voltage of 100 kV (GnPC and GnPH), and a Philips CM20 with EDS system at an acceleration voltage of 200 kV (GnPH). Particles were previously dispersed in a dilute butyl alcohol solution (1.2 g·L^−1^), submitted to ultrasonication for two hours, deposited on a carbon coated copper grid, and dried under a lamp.

### 2.4. Characterization of Nanocomposites

PP/GnP nanocomposite sections with a thickness of 10 µm were cut perpendicularly and parallel to the flow direction with a Leitz 1401 microtome at room temperature, using glass knives with an angle of 45°. Optical micrographs were acquired using a Leica DFC 280 digital camera coupled to a BH2 Olympus microscope. For each composite, at least 12 micrographs were analyzed using ImageJ^®^ software (open source), leading to an investigated total area of 4.2 mm^2^. The area of the agglomerates larger than 5 µm^2^ was measured, implying that smaller agglomerate areas were not accounted for in the statistical study. The total number of agglomerates measured for the analysis (*N*) was identified. The average agglomerate area (*A*_av_) was calculated, as well as its variance within a confidence interval of 95%. The parameters used to describe the degree of GnP dispersion were the agglomerate area ratio (*A*_r_) and the number of agglomerates per unit area (*N*_A_). *A*_r_ is defined as the ratio between the sum of the areas of all agglomerates (∑GnP) and the total composite area analyzed (*A*_T_), as indicated in Equation (1),

(1) $$A_{r}=\frac{\sum \mathrm{GnP}}{A_{T}}\times 100$$

thus providing a convenient overall dispersion index. A cumulative distribution of the agglomerate areas can be defined as

(2) $$CA_{j}=\frac{\sum_{i=1}^{j}A_{i}}{\sum \mathrm{GnP}}\times 100$$

where ∑*A*_i_ is the summation of the areas of the individual agglomerates *i* in ascending area order. *CA*_j_ provides detailed information about the size distribution of the surviving agglomerates. For example, it allows for the comparison of the agglomerate area populations of the composites collected along the mixer.

TEM analysis of the GnPC composite was carried out in a Libra 120 microscope (Carl Zeiss Microscopy GmbH, Oberkochen, Germany) at 120 kV. The specimens were cut into approx. 80 nm thin sections with a diamond knife (DiATOME AG, Biel, Switzerland) using an Ultracut UC6 ultramicrotome (Leica Microsystems GmbH, Wetzlar, Germany).

The alternate current (AC) electrical conductivity of PP/GnP nanocomposites was measured with a Quadtech (Sussex, WI, USA) 1920 Precision LCR meter directly on the disks collected from the last flow channel, near to the mixer outlet. Experiments were performed at room temperature at a voltage of 1 V, in the frequency range of 5 × 10^2^–1 × 10^6^ Hz. Circular contacts with a diameter of 6 mm and a thickness of 50 nm were produced by magnetron sputtering of gold/palladium.

## 3. Results and Discussion

### 3.1. Graphite Nanoplates Size and Morphology

**Figure 2:**
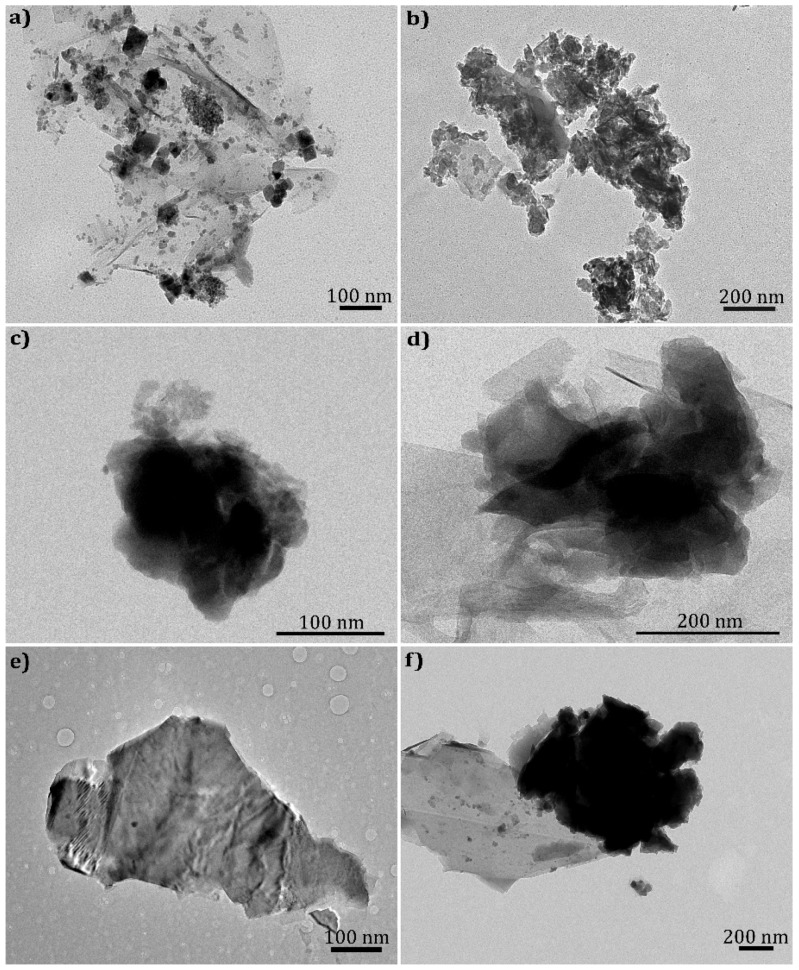
TEM micrographs of as-received GnPC, GnPM, and GnPH (**a**,**c**,**e**) and *f*GnPC-PP, *f*GnPM-PP, and *f*GnPH-PP (**b**,**d**,**f**).

Graphite nanoplates with different widths and thicknesses (see Table 1) were supplied in powder form. Typically, their primary nanoparticles form agglomerates with high cohesive strength, which has practical consequences on the rate and intensity of dispersion in the polymer melt during nanocomposite processing. The average area of the as-received GnP and *f*GnP-PP primary nanoparticle agglomerates, and the significance within a 95% confidence interval, was calculated and is presented in Table 2. Different average areas were measured for each GnP type, and it was consistently observed that the nanoparticles functionalized with PP-*g*-MA formed larger agglomerates. Figure 2 depicts TEM micrographs of all powders used in this work, showing their flake morphology. As-received GnPC is composed of smaller flakes compared to GnPM and GnPH. After surface chemical modification, the morphology of all graphite nanoplates seems to be maintained, despite the increase in size, suggesting that the method carried out under the conditions described does not induce damage of the GnP structure.

### 3.2. Dispersion of the Graphite Nanoplates in Polypropylene

**Figure 3:**
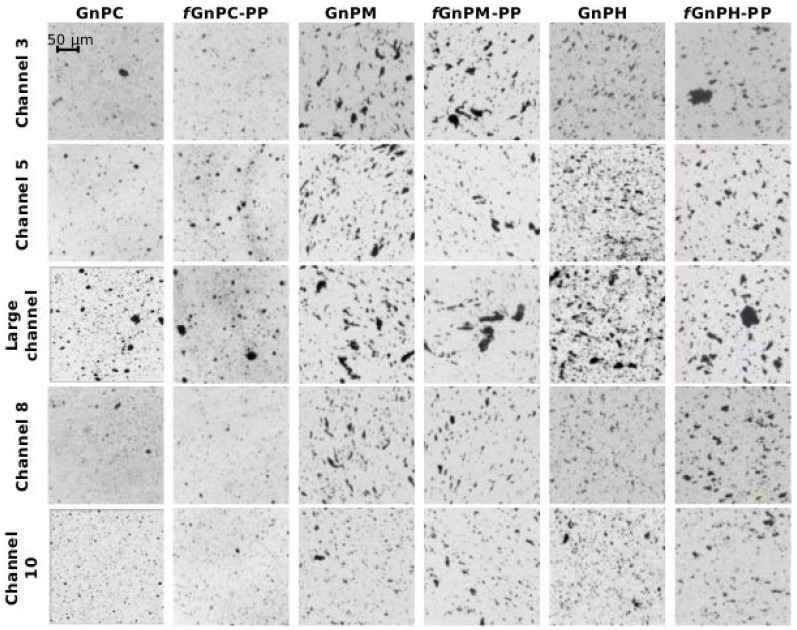
Optical micrographs of samples of PP nanocomposites containing GnP and *f*GnP-PP collected from a prototype small-scale extensional mixer.

**Figure 4:**
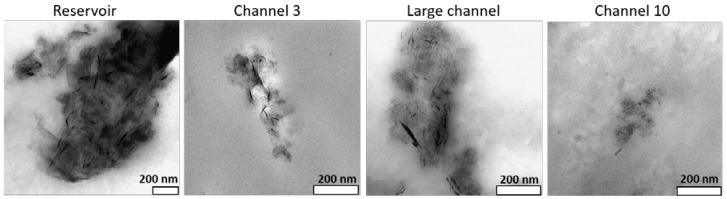
TEM micrographs of the GnPC composite collected along the extensional mixer.

Optical micrographs illustrating the morphology of the GnP agglomerates for as-received and functionalized GnP in the PP nanocomposites are presented in Figure 3. The images depict the changes in GnP agglomerate size as the melt progresses along the extensional mixer, from the reservoir of the capillary rheometer to the exit of the mixer. In channel 3, the composite has been subjected to three converging/diverging flows. Channel 5 corresponds to the fifth and last converging/diverging sequence of the first mixing zone, before the material advances in the larger cylindrical channel into channel 8. The melt was again subjected to three converging/diverging flows. Finally, channel 10 induces the last converging/diverging sequence prior to the material exiting the mixer (thus, channels 8 and 10 belong to the second mixing zone). Differences in size of the agglomerates at the various locations along the mixer are readily identifiable, with a notorious increase in size in the long channel. Differences in the behavior of the various grades of GnP are also evident. TEM micrographs of the GnPC composites presented in Figure 4 illustrate the dispersion effect as observed at high magnification. At the nanometer scale, larger and denser agglomerates are observed at the reservoir, becoming smaller after channel 3, reforming inside the large channel (although appearing to be less dense), and decreasing in size again after passing channel 10.

The results of the OM analysis are summarized in Table 3, which presents, for the same locations of the mixer in Figure 3, and also at the reservoir of the rheometer, the average agglomerate areas (*A*_av_) and their significance within a 95% confidence interval, the number of agglomerates per unit area of the composite sections analyzed (*N*_A_), and the total number of agglomerates measured for the analysis (*N*). Interestingly, the *A*_av_ of the as-received and functionalized GnP agglomerates in the composite collected from the reservoir are approximately three times smaller than the corresponding powder average area, for all GnP types (Table 2). This is indicative that pressing the powder and melting the polymer by heat conduction from the barrel of the rheometer and flow under low shear rate create enough thermomechanical stresses to initiate GnP agglomerate breakage. From the reservoir to channel 5, a decrease in GnP size, as well as in the number of agglomerates per unit area of composite cross-section, is evident for all GnP grades and functionalized equivalents. At the large cylindrical channel, where the average shear rate is very low (~0.3 s^−1^) and the local residence time is substantial, the GnP agglomerates reorganize and undergo re-agglomeration. The area of the agglomerates rises to values close to those of the agglomerates present inside the reservoir of the rheometer. Simultaneously, the number of agglomerates per unit area, *N*_A_, increases relative to those present in channel 5, although they are less than those in the reservoir, for all GnP types. This could suggest that the morphology/cohesiveness of the new agglomerates might be distinct from those in the melt in the reservoir. In the second mixing zone (from channel 6 to channel 10), the average size of the agglomerates reduces, demonstrating again that increasing the shear rate resets dispersion. The *A*_av_ reached in channel 10 is similar to that achieved in channel 5. These results are globally in line with those obtained for PP/MWCNT composites being processed and reprocessed under identical operating conditions [38,39].

**Figure 5:**
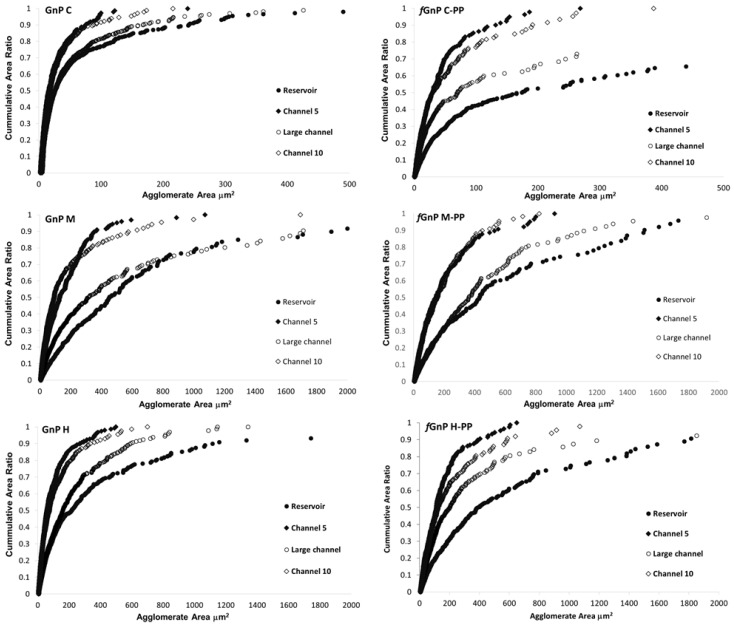
Representation of the cumulative agglomerate area distribution measured for the PP/GnP and PP/*f*GnP-PP composites collected along the mixer.

Figure 5 displays the cumulative distribution of the agglomerate areas, *CA*_i_, for the as-received and chemically modified particles. For the sake of clarity, only the cumulative distributions of the agglomerate areas of the composites in the reservoir, channel 5 (end of first mixing zone), large channel (where nanoparticle re-agglomeration was observed), and channel 10 (end of second mixing zone) are represented. The composites with all GnP types, with and without functionalization, present a wide distribution of agglomerate areas with large agglomerates in the reservoir, while the narrower distributions with smaller agglomerates are mostly observed in channel 5. The large channel allows substantial re-agglomeration, reforming large agglomerates and presenting a wide distribution of agglomerate areas, although smaller than measured in the reservoir. Finally, in channel 10, at the exit of the second mixing zone, the agglomerates are smaller and form a narrow distribution, approaching that observed in channel 5. GnPC and *f*GnPC-PP form considerably smaller agglomerates compared to GnPM, *f*GnPM-PP, GnPH, and *f*GnPH-PP. The cumulative distributions of the agglomerates formed by GnPM and GnPH are of comparable width, with agglomerate sizes of similar magnitude. Functionalization of the nanoparticles did not significantly change the range of agglomerate sizes formed.

**Figure 6:**
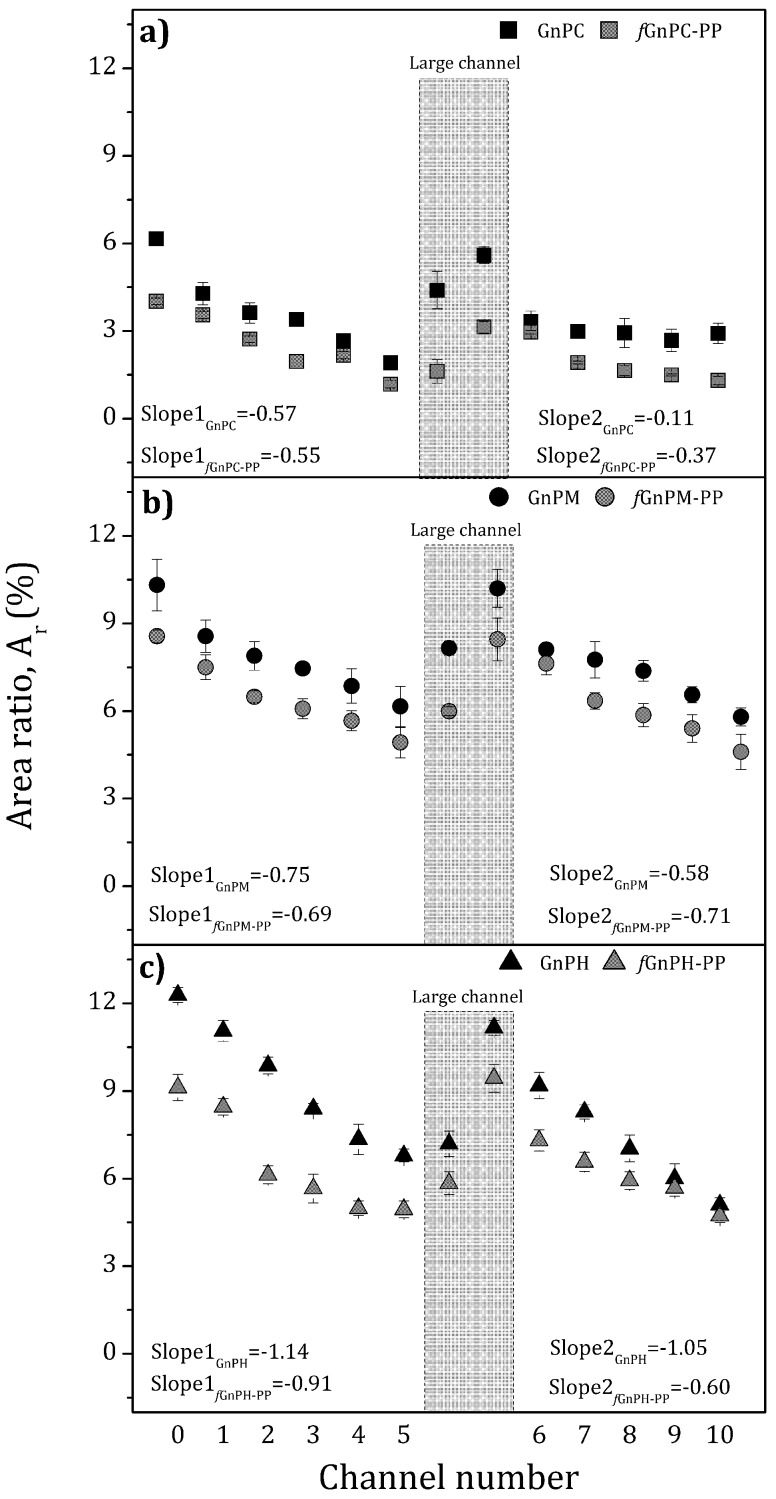
Evolution of dispersion (in terms of Area Ratio, *A*_r_) along the length of the extensional mixer of GnP and *f*GnP-PP in a PP melt matrix.

The state of dispersion of carbon nanoparticles is commonly associated to the inverse of *A*_r_, i.e., complete dispersion at the microscopic level corresponds to *A*_r_ = 0, while composites containing a large number of agglomerates and/or large agglomerate areas present high values of *A_r_*. The *A*_r_ values obtained for GnPC, GnP M, and GnPH and functionalized equivalents, are depicted in Figure 6. In all cases, *A*_r_ shows a consistent, almost linear, decrease from the reservoir to the end of the first mixing section (channel 5). Two samples from the large channel were collected and analyzed, one from a location near to its entrance, the other near to the exit. Figure 6 clearly demonstrates that a systematic increase of *A*_r_ along the relaxation chamber takes place, showing evidence for GnP or *f*GnP-PP re-agglomeration. The values of *A*_r_ attained approach those measured upstream in the reservoir (channel 0). However, as noted above, these new agglomerates could have distinct cohesiveness or shape, as these characteristics do not influence the values of *A*_r_. When the composite melt is resubmitted between channels 6 to 10 to the shear rate conditions imposed along the first mixing zone, *A*_r_ decreases and reaches values similar to those attained at the outlet of channel 5.

The rate of *A*_r_ evolution along the equipment varies with GnP type, as perceived from the linear slopes indicated in Figure 6. These observations may be analyzed in light of the rupture and erosion dispersion mechanisms of agglomerated particles in melts postulated by Manas-Zloczower and co-workers [40,41]. These authors postulated that the route to dispersion depends on the magnitude of a fragmentation number, F_a_, and on the on the probability for break-up, P_break_. F_a_ balances the hydrodynamic stresses against the cohesive strength of the agglomerate. As F_a_ increases progressively above 1, erosion (2 ≤ F_a_ < 5) or rupture (F_a_ ≥ 5) will become gradually predominant. The quasi-linear progression of dispersion along the mixer seems to indicate that a single dispersion route is pursued, probably rupture, as erosion is known to be a slow process [42]. P_break_ is proportional to the residence time and agglomerate area. Thus, at constant stress, which is the case of the experiments performed (where the total pressure drop was kept approximately constant), the smaller the agglomerate, the longer the exposure time required to break it. As seen in Figure 6, the slopes follow the rank GnPH > GnPM > GnPC, consistent with the increase in the area-to-volume ratio for these nanoparticles which, according to the particle dimensions indicated by the producer, is approximately 0.13, 0.33, and 1.0 for GnPH, GnPM, and GnPC, respectively. Thus, Figure 6 shows that the smaller the nanoparticle (and thus, the larger its area-to-volume ratio), the slower is its kinetics of dispersion.

The discussion above remains valid for the various *f*GnP-PP. Nevertheless, two further observations seem relevant: (i) The overall dispersion level reached for *f*GnP-PP is systematically higher compared to as-received GnP; (ii) chemical modification of the GnP surface did not significantly change the re-agglomeration behavior.

### 3.3. Electrical Conductivity of the Nanocomposites

**Figure 7:**
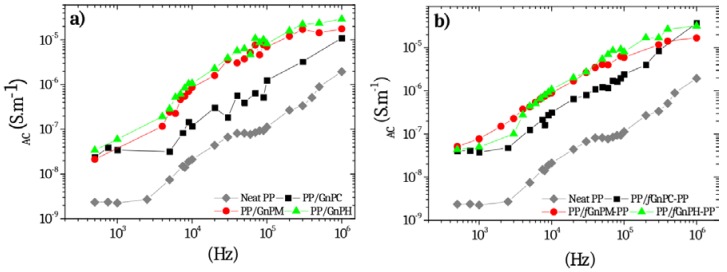
AC electrical conductivity of PP nanocomposites with 2 wt % of (**a**) as-received GnP and (**b**) *f*GnP-PP.

The effects of size and surface chemistry of graphite nanoplates on the AC electrical conductivity of PP nanocomposites are shown in Figure 7a,b, respectively. PP shows a typical behavior of a dielectric material, the electrical conductivity increasing with the frequency in a logarithmic scale. The incorporation of 2 wt % of as-received GnP and *f*GnP-PP increased slightly the electrical conductivity of PP from 10^−9^ to 10^−8^ S/m at 1 KHz. However, nanocomposites still exhibit a frequency-dependent behavior, showing that the formation of an interconnected conductive network was not achieved. As shown elsewhere [3,11,35], even at filler loadings as high as 10 wt %, percolation may not be achieved. No significant differences on the AC conductivity of PP composites containing 2 wt % graphite nanoplates with different widths and thicknesses were found, however, the AC conductivity is systematically higher for the composites with larger GnP flake.

## 4. Conclusions

The dispersion of different GnP grades in a PP melt was carried out in a prototype extensional mixer under constant hydrodynamic stress conditions, in order to investigate the influence of the GnP flake size on dispersion rate and stability. GnP flakes with larger surface area (smaller flake size) and higher bulk density presented smaller agglomerate size in the powder form and final smaller average agglomerate area, as measured by OM in the PP composite, in contrast with the GnP with larger flakes and lower bulk density.

The degree of dispersion, as defined by the agglomerate area ratio, *A*_r_, increased linearly along the mixer length, irrespective of the GnP morphology and bulk density; however, the dispersion rate along the mixer was lower for the smaller flakes with higher bulk density. Once a certain dispersion level is attained, if the composite is made to flow at sufficiently low shear rates during enough time, an increase of the *A*_r_ was observed for all the GnP types, indicating re-agglomeration of GnP, subjecting the composite to the same flow conditions as before induces dispersion, although at a slightly lower rate compared to the first mixing zone, which may be indicative of differences in the agglomerate cohesive forces before and after re-agglomeration.

Functionalization by covalent bonding polymer molecules to the GnP surface should increase the GnP/PP compatibility and interfacial strength; the functionalized GnP showed similar dispersion effects as observed for the pristine GnP, however, presenting slightly higher dispersion levels.

The 2 wt % GnP composites presented dielectric behavior, but the AC conductivity was systematically higher for the composites with larger GnP flakes (with and without functionalization).

## Figures and Tables

**Table 1:** Properties of the as-received graphite nanoplates.

xGnP	Carbon purity (%)	Bulk density	Density	Width (µm)	Thickness (nm)	Surface area (m^2^·g^−1^)	Electrical conductivity in plane/perpendicular to surface (S·m^−1^)
xGnP Grade C^®^	98.0	0.20–0.40	2.0–2.25	1–2	2	750	Not available
xGnP Grade M^®^	>99.5	0.03–0.10	2.2	15	6-8	120–150	10^7^/10^2^
xGnP Grade H^®^	>99.5	0.03–0.10	2.2	5	15	60–80	10^7^/10^2^

**Table 2:** Average area of the GnP primary nanoparticle agglomerates and significance within a 95% confidence interval.

GnP powder	Average area of the GnP powders (µm^2^)
GnPC	45 ± 4
*f*GnPC-PP	71 ± 12
GnPM	326 ± 34
*f*GnPM-PP	758 ± 123
GnPH	194 ± 11
*f*GnPH-PP	289 ± 17

**Table 3:** Characterization of dispersion and re-agglomeration of PP nanocomposites containing 2 wt % of as-received GnP and *f*GnP-PP during flow in a prototype small-scale extensional mixer.

Location in extensional mixer	*A*_av_ (µm^2^) *	*N*_A_ (mm^−2^)	*N*	*A*_av_ (µm^2^) *	*N*_A_ (mm^−2^)	*N*
	PP/GnPC			PP/*f*GnPC-PP		
Reservoir	15.0 ± 0.9	240.7	4289	24 ± 8	2000.3	1828
Channel 3	14 ± 2	152.2	862	12 ± 2	275.4	1722
Channel 5	10.7 ± 0.7	74.93	1650	10 ± 1	131.5	1237
Large channel	14.6 ± 0.8	167.4	3989	14 ± 4	634.0	1170
Channel 8	18 ± 2	139.5	1046	12 ± 2	279.1	1436
Channel 10	14.6 ± 0.9	88.48	1298	10 ± 1	191.3	1335
	PP/GnPM			PP/*f*GnPM-PP		
Reservoir	131 ± 19	1895.1	827	120 ± 19	1510.4	744
Channel 3	63 ± 6	644.8	1243	72 ± 8	571.2	880
Channel 5	51 ± 4	367.4	1268	56 ± 6	530.1	920
Large channel	83 ± 4	1712.2	1288	110 ± 14	1113.0	800
Channel 8	47 ± 4	543.4	1626	64 ± 8	745.2	951
Channel 10	40 ± 4	575.1	1511	60 ± 6	498.0	805
	PP/GnPH			PP/*f*GnPH-PP		
Reservoir	65 ± 7	1157.6	1975	110 ± 17	1814.9	975
Channel 3	38 ± 3	297.4	2331	57 ± 6	476.4	901
Channel 5	32 ± 2	267.7	2197	50 ± 5	388.4	994
Large channel	56 ± 5	606.0	2067	71 ± 11	1182.8	1031
Channel 8	38 ± 3	384.6	1953	62 ± 8	823.5	1034
Channel 10	34 ± 3	360.4	1582	55 ± 7	588.2	862

* Average agglomerate area considering the *N* agglomerates measured with a confidence interval of 95%.
